# Supplementary material for: A Likelihood Approach for Real-Time Calibration of Stochastic Compartmental Epidemic Models
Source: PLoS Comput Biol. 2017 Jan 17;13(1):e1005257. doi: 10.1371/journal.pcbi.1005257 (PMC5240920; doi:10.1371/journal.pcbi.1005257)
Supplement: S1 File — (TAR.GZ) [file pcbi.1005257.s014.tar.gz › HSPH_Online-SI-Revision/EnKF07/n1-extreme/n1-extreme_EnKF_table-4.pdf]

1	R0	{2.95019, 14.2854}	{2.63772, 7.10533}	{{2.1613, 2.50664}, {3., 12.9816}}	{12.5501, 50.5944}	inf	56.	221.908	{58.3397, 359.681}	303.584	0.53472	newly	31.	225.25	{63., 559.}	631.	0.731966	Reff	2.70237	0.823461	{0.229493, 1.56209}	69.5282	ar	924.	1552.27	{1061., 2190.}	71.3561
2	R0	{2.67565, 16.946}	{2.57584, 5.15874}	{{2.12979, 0.63827}, {3., 7.06717}}	{19.0044, 69.5558}	inf	194.	48.8002	{12.381, 94.2073}	78.3076	0.773822	newly	79.	66.65	{12., 159.}	90.9747	0.50079	Reff	1.69101	0.549969	{0.142237, 0.916298}	70.1676	ar	926.	1724.12	{1635., 2144.}	89.3715
3	R0	{2.40066, 16.6543}	{2.2275, 7.10702}	{{1.23656, 2.70463}, {3., 14.4209}}	{29.5472, 59.7724}	inf	86.	150.322	{36.6135, 348.267}	102.898	0.268283	newly	43.	135.39	{32., 324.}	227.326	0.388503	Reff	2.02856	0.524986	{0.109792, 1.26099}	74.8922	ar	916.	1598.71	{1035., 2096.}	77.8657
4	R0	{1.9294, 15.4361}	{2.01244, 5.14426}	{{0.24047, 1.96588}, {3., 7.485}}	{51.1084, 67.751}	inf	96.	93.4916	{10.6877, 169.899}	70.8048	0.37961	newly	51.	92.75	{11., 157.}	123.471	0.358964	Reff	1.6207	0.395257	{0.03266, 0.593695}	75.6119	ar	796.	1564.16	{597., 1786.}	105.432
5	R0	{2.92859, 19.5393}	{2.81598, 7.66999}	{{2.40461, 3.63707}, {3., 15.6491}}	{7.06385, 60.7458}	inf	172.	37.4392	{14.5918, 30.6056}	90.0917	0.971154	newly	68.	42.16	{5., 39.}	106.059	0.735915	Reff	1.94751	0.511724	{0.173441, 0.599314}	74.7583	ar	927.	1690.3	{1550., 2105.}	82.3409
6	R0	{1.79622, 12.3148}	{2.52127, 6.59594}	{{1.65364, 3.45813}, {3., 9.63071}}	{47.875, 51.4468}	inf	52.	43.7316	{7.87325, 54.5623}	79.6347	0.426197	newly	27.	49.21	{4., 45.}	142.185	0.355072	Reff	1.38489	0.443965	{0.0346086, 0.638865}	70.0532	ar	734.	1800.03	{1662., 1948.}	145.236
7	R0	{2.45855, 17.1776}	{2.52105, 6.63643}	{{1.89814, 1.50987}, {3., 12.5842}}	{19.7703, 62.1498}	inf	118.	205.115	{78.9425, 591.122}	94.1382	0.257611	newly	59.	221.97	{45., 709.}	287.678	0.453968	Reff	1.87833	0.702193	{0.211548, 1.5822}	64.3673	ar	871.	1624.55	{1052., 2293.}	87.3858
8	R0	{2.20009, 15.519}	{2.68442, 9.45216}	{{2.2663, 3.78506}, {3., 17.0883}}	{27.6639, 43.8383}	inf	146.	60.3038	{12.498, 54.5848}	86.8934	0.74486	newly	70.	56.85	{8., 55.}	111.243	0.652096	Reff	1.44546	0.556789	{0.230536, 0.796181}	67.1432	ar	839.	1795.65	{1642., 2057.}	114.023
9	R0	{2.06493, 9.6414}	{2.61772, 7.10219}	{{2.15593, 2.55184}, {3., 12.7312}}	{32.2814, 38.5831}	inf	38.	216.057	{69.2809, 361.076}	473.681	0.691761	newly	41.	217.59	{67., 554.}	436.024	0.623535	Reff	1.89148	0.822142	{0.237099, 1.50461}	58.7164	ar	777.	1540.11	{1076., 2064.}	101.188
10	R0	{2.60775, 19.6936}	{2.65909, 9.29024}	{{2.24048, 5.85113}, {3., 16.6111}}	{17.2519, 52.8261}	inf	154.	41.66	{4.4018, 59.128}	85.2914	0.889931	newly	62.	35.13	{2., 38.}	92.5968	0.739489	Reff	1.84629	0.553408	{0.0936102, 1.01534}	72.1488	ar	889.	1767.16	{1630., 1878.}	98.7807
11	R0	{1.99168, 8.13555}	{2.51737, 6.7395}	{{1.89637, 1.80909}, {3., 12.7559}}	{32.6163, 48.0509}	inf	65.	208.579	{83.719, 589.018}	226.323	0.42492	newly	78.	231.66	{58., 719.}	208.128	0.353603	Reff	1.67899	0.709602	{0.183061, 1.58124}	61.8673	ar	772.	1609.03	{982., 2321.}	110.911
12	R0	{1.79304, 9.18941}	{2.5461, 6.99443}	{{2.12241, 2.52847}, {3., 12.7584}}	{48.8291, 38.3012}	inf	28.	210.355	{59.3218, 378.461}	654.789	0.781487	newly	33.	218.74	{60., 559.}	567.03	0.681858	Reff	1.69442	0.82294	{0.225926, 1.56941}	55.9134	ar	725.	1558.33	{1063., 2085.}	117.204
13	R0	{1.84016, 18.3431}	{2.62416, 9.74178}	{{2.24117, 4.67902}, {3., 16.8233}}	{52.5751, 47.242}	inf	89.	57.2506	{10.311, 55.7683}	89.5038	0.522246	newly	37.	50.85	{7., 36.}	128.946	0.397465	Reff	1.48685	0.569843	{0.13846, 0.917588}	65.775	ar	762.	1798.15	{1648., 2167.}	136.644
14	R0	{1.94221, 9.85971}	{1.97334, 5.05621}	{{0.178183, 1.7547}, {3., 7.28614}}	{53.9086, 52.7411}	inf	111.	124.133	{18.7402, 294.121}	83.5945	0.397393	newly	83.	142.61	{17., 307.}	130.47	0.424479	Reff	1.44306	0.405998	{0.0260085, 0.630482}	75.9138	ar	805.	1691.83	{1455., 2232.}	113.914
15	R0	{1.78578, 12.0541}	{2.65655, 6.20468}	{{1.78435, 2.61865}, {3., 10.9937}}	{50.7648, 57.826}	inf	101.	31.5444	{4.21512, 44.9679}	85.0452	0.818636	newly	70.	34.19	{3., 68.}	82.9571	0.716516	Reff	1.39648	0.540376	{0.119009, 1.3389}	64.7225	ar	732.	1727.29	{1610., 1940.}	135.969
16	R0	{1.94463, 15.6503}	{2.53228, 6.44768}	{{1.85645, 2.08906}, {3., 12.3885}}	{34.6208, 59.976}	inf	94.	200.928	{64.6337, 588.747}	130.628	0.332118	newly	42.	208.33	{43., 472.}	404.69	0.574727	Reff	1.60626	0.667822	{0.192787, 1.17222}	62.4807	ar	757.	1582.02	{1146., 2307.}	110.959
17	R0	{2.08901, 16.4314}	{2.51196, 5.63572}	{{2.13045, 0.577798}, {3., 9.97108}}	{26.1475, 66.0114}	inf	110.	71.9306	{10.6636, 141.901}	82.3066	0.537408	newly	50.	120.18	{10., 299.}	207.24	0.439441	Reff	1.69628	0.6632	{0.130853, 2.12832}	67.1535	ar	826.	1828.45	{1617., 2512.}	124.938
18	R0	{2.76419, 18.56}	{2.56012, 6.97175}	{{1.8915, 1.70147}, {3., 12.9751}}	{16.5958, 62.7856}	inf	102.	187.223	{32.7569, 346.06}	107.874	0.319222	newly	42.	179.18	{26., 382.}	342.048	0.52743	Reff	2.30257	0.61834	{0.125682, 1.0808}	73.4912	ar	920.	1532.42	{1083., 2105.}	70.3543
19	R0	{2.3821, 17.1399}	{2.54726, 7.25037}	{{2.12576, 2.59745}, {3., 12.7158}}	{20.6971, 57.6989}	inf	70.	227.593	{77.7249, 378.976}	232.714	0.463255	newly	39.	217.55	{68., 387.}	462.538	0.646755	Reff	2.06528	0.841302	{0.241106, 1.54037}	60.3303	ar	883.	1572.18	{1047., 2163.}	80.2084
20	R0	{2.78314, 19.6127}	{2.65434, 7.00579}	{{2.24733, 0.224898}, {3., 16.1421}}	{15.0364, 64.3047}	inf	270.	33.7157	{3.48933, 51.7799}	88.3604	1.09527	newly	81.	36.25	{4., 49.}	82.1111	0.697196	Reff	1.35817	0.447205	{0.101751, 0.755282}	69.6851	ar	943.	1722.14	{1616., 1822.}	82.6235
21	R0	{1.85124, 14.6794}	{2.58259, 5.6605}	{{2.12786, 0.670338}, {3., 9.59654}}	{42.6627, 62.1308}	inf	54.	57.0222	{10.107, 102.77}	76.1534	0.332658	newly	27.	91.1	{9., 272.}	268.593	0.377638	Reff	1.61058	0.597336	{0.122014, 1.30914}	70.3908	ar	730.	1770.14	{1592., 2259.}	143.844
22	R0	{2.1028, 14.9852}	{1.99924, 4.72891}	{{0.150426, 1.73321}, {3., 7.62009}}	{47.9522, 69.2443}	inf	146.	112.907	{18.097, 256.07}	71.7635	0.436091	newly	80.	127.81	{15., 300.}	116.238	0.374835	Reff	1.46565	0.385855	{0.0236175, 0.639786}	75.0975	ar	840.	1633.13	{878., 2031.}	100.365
23	R0	{1.86601, 10.2157}	{2.56964, 7.40826}	{{1.8492, 1.80172}, {3., 13.2722}}	{40.7458, 49.2085}	inf	53.	186.946	{66.498, 325.129}	259.317	0.481608	newly	50.	170.41	{40., 346.}	250.9	0.441199	Reff	1.64022	0.619868	{0.105245, 1.02427}	65.664	ar	747.	1521.96	{1053., 2093.}	105.349
24	R0	{2.34032, 10.0992}	{2.53409, 7.04098}	{{2.12601, 2.08333}, {3., 12.9187}}	{22.3643, 40.7619}	inf	45.	220.742	{63.7678, 364.948}	400.937	0.652981	newly	41.	222.04	{57., 547.}	451.561	0.65275	Reff	2.12969	0.827439	{0.204108, 1.57483}	61.888	ar	887.	1560.1	{1022., 2228.}	79.4183
25	R0	{1.91548, 15.3401}	{2.00623, 5.26982}	{{0.199436, 1.92833}, {3., 6.31831}}	{52.1789, 67.0152}	inf	62.	96.5307	{14.5133, 184.53}	96.5499	0.328526	newly	31.	94.47	{11., 152.}	230.226	0.421631	Reff	1.66455	0.373801	{0.0323431, 0.609905}	77.5434	ar	765.	1572.96	{849., 1772.}	112.11
26	R0	{2.75283, 10.6721}	{2.50575, 6.59608}	{{1.76792, 1.67948}, {3., 12.7197}}	{18.3136, 50.8479}	inf	42.	190.259	{82.2088, 327.649}	357.826	0.586383	newly	48.	199.54	{71., 362.}	321.542	0.527881	Reff	2.52985	0.641105	{0.210937, 1.05088}	74.6584	ar	926.	1561.19	{1130., 2042.}	70.0594
27	R0	{2.08367, 19.7849}	{2.33618, 5.03537}	{{0.625494, 2.13733}, {3., 10.4787}}	{45.8943, 74.5494}	inf	122.	55.8729	{3.55529, 51.5297}	93.1941	0.801139	newly	56.	58.51	{2., 59.}	106.661	0.546885	Reff	1.4669	0.419909	{0.0984152, 0.621668}	74.2972	ar	833.	1811.54	{1668., 1894.}	117.472
28	R0	{2.64334, 17.1765}	{2.26943, 6.76903}	{{1.23442, 2.78716}, {3., 12.8055}}	{25.9134, 62.0451}	inf	150.	151.239	{25.5721, 375.047}	75.3663	0.355347	newly	88.	141.87	{18., 326.}	109.761	0.322481	Reff	1.90056	0.492951	{0.0995831, 1.21789}	75.13	ar	934.	1598.74	{1033., 2072.}	74.334
29	R0	{2.87008, 13.3872}	{2.53866, 6.6143}	{{1.60932, 4.21524}, {3., 8.83521}}	{17.1604, 53.1252}	inf	74.	47.2915	{5.75883, 55.5015}	82.5134	0.538348	newly	61.	51.76	{5., 48.}	102.59	0.578167	Reff	2.46827	0.439693	{0.034364, 0.707858}	82.2592	ar	941.	1786.33	{1645., 2064.}	91.0255
30	R0	{2.06105, 19.6275}	{2.79627, 5.02614}	{{2.41368, 2.47941}, {3., 6.43855}}	{35.6721, 74.3924}	inf	138.	53.7129	{5.78929, 47.5177}	95.8168	0.904066	newly	44.	64.19	{4., 60.}	148.432	0.545084	Reff	1.05732	0.498533	{0.201474, 0.75561}	60.0893	ar	747.	1725.52	{1583., 2041.}	130.993
31	R0	{2.30764, 13.435}	{2.56586, 6.70475}	{{1.87458, 1.91844}, {3., 12.8354}}	{23.7227, 54.1185}	inf	103.	174.02	{77.0378, 304.597}	86.9735	0.265821	newly	60.	172.43	{39., 331.}	201.917	0.412395	Reff	1.88303	0.629665	{0.204449, 1.03212}	67.3416	ar	877.	1558.6	{1150., 2111.}	79.5143
32	R0	{2.30565, 11.9237}	{2.54263, 6.95485}	{{1.85445, 0.87085}, {3., 13.1331}}	{23.9915, 51.8537}	inf	62.	196.604	{61.9487, 358.177}	229.475	0.461011	newly	41.	182.66	{37., 400.}	357.22	0.541332	Reff	1.97825	0.597848	{0.105189, 1.05081}	70.8002	ar	853.	1557.86	{1016., 2298.}	87.2216
33	R0	{2.24031, 18.582}	{2.42885, 7.25766}	{{1.60817, 3.79779}, {3., 14.1818}}	{29.9389, 60.9425}	inf	83.	56.3952	{4.85212, 78.3309}	94.1633	0.628792	newly	35.	56.01	{3., 104.}	142.829	0.479413	Reff	1.90874	0.477796	{0.0378582, 0.752945}	75.8187	ar	864.	1806.39	{1632., 2257.}	110.247
34	R0	{2.33306, 15.9759}	{2.25736, 7.09292}	{{1.25562, 3.36072}, {3., 13.6655}}	{29.5814, 58.8918}	inf	138.	136.832	{27.9815, 299.978}	67.6284	0.332934	newly	70.	120.99	{22., 241.}	109.986	0.303296	Reff	1.80112	0.461622	{0.0699118, 0.851919}	76.4502	ar	854.	1591.16	{1034., 2022.}	87.7963
35	R0	{2.48357, 18.3573}	{2.55332, 6.46598}	{{1.92837, 2.34098}, {3., 12.6555}}	{19.5961, 64.9329}	inf	114.	195.18	{35.5896, 588.008}	97.6533	0.289197	newly	58.	195.15	{35., 480.}	252.431	0.442596	Reff	1.98686	0.628252	{0.176559, 1.04243}	69.3934	ar	835.	1559.22	{1077., 2139.}	87.9066
36	R0	{2.59548, 19.9046}	{2.47679, 5.79949}	{{2.10642, 0.635585}, {3., 10.1854}}	{20.6621, 70.8636}	inf	110.	73.5687	{13.4419, 132.65}	75.3486	0.475206	newly	53.	114.73	{15., 255.}	178.698	0.405243	Reff	2.02967	0.660249	{0.102786, 2.12758}	68.7243	ar	863.	1860.04	{1644., 2561.}	118.153
37	R0	{2.29641, 16.9868}	{2.25312, 5.73674}	{{0.864182, 0.477094}, {3., 10.5501}}	{37.3597, 66.3585}	inf	149.	47.0301	{7.52217, 94.5336}	85.7795	0.844612	newly	59.	55.67	{4., 166.}	109.305	0.625638	Reff	1.61208	0.431047	{0.0910629, 0.879185}	74.0379	ar	877.	1756.21	{1604., 2135.}	100.252
38	R0	{2.65743, 15.4569}	{2.59682, 5.26342}	{{2.12862, 0.60185}, {3., 10.2475}}	{17.4924, 65.9477}	inf	119.	53.658	{12.1233, 98.0277}	69.47	0.540374	newly	62.	91.57	{12., 206.}	128.177	0.435054	Reff	1.96118	0.623433	{0.14928, 1.20505}	70.5573	ar	897.	1823.91	{1647., 2371.}	106.358
39	R0	{2.67953, 14.5132}	{2.5083, 6.58938}	{{1.78821, 1.6127}, {3., 12.7335}}	{18.8286, 57.8249}	inf	36.	191.21	{78.4188, 327.735}	435.712	0.651563	newly	28.	200.45	{68., 367.}	619.536	0.744563	Reff	2.47857	0.637332	{0.202755, 1.02982}	74.2862	ar	894.	1562.82	{1113., 2076.}	76.1163
40	R0	{1.94594, 11.928}	{2.09007, 4.95006}	{{0.193504, 1.48501}, {3., 7.42084}}	{50.9494, 60.0934}	inf	81.	124.163	{18.9257, 271.272}	95.9391	0.327537	newly	45.	149.72	{14., 402.}	264.489	0.471471	Reff	1.35827	0.432272	{0.0209618, 0.670827}	75.4658	ar	796.	1722.38	{1497., 2194.}	118.06
41	R0	{1.66297, 10.0047}	{2.70018, 6.89527}	{{1.8025, 2.96786}, {3., 15.0327}}	{63.9903, 52.7337}	inf	49.	43.9783	{5.45814, 53.957}	96.4403	0.510615	newly	37.	42.68	{4., 51.}	114.108	0.482604	Reff	1.32539	0.483104	{0.0947014, 0.825776}	66.4467	ar	716.	1740.76	{1609., 1904.}	143.123
42	R0	{2.91568, 16.4453}	{2.56415, 7.29479}	{{2.13148, 2.59147}, {3., 12.6423}}	{15.2956, 55.6736}	inf	83.	227.964	{62.6504, 391.878}	185.275	0.401245	newly	52.	214.7	{63., 417.}	318.346	0.521376	Reff	2.50748	0.809308	{0.237382, 1.51346}	67.7243	ar	919.	1560.97	{1075., 2209.}	72.5234
43	R0	{2.55898, 15.8889}	{2.55366, 6.76805}	{{1.87553, 1.53821}, {3., 12.7467}}	{19.3428, 59.3115}	inf	88.	184.667	{77.6015, 322.576}	120.459	0.305016	newly	64.	192.2	{57., 352.}	210.844	0.408435	Reff	2.22119	0.656399	{0.200194, 1.04812}	70.703	ar	904.	1579.6	{1142., 2113.}	75.9956
44	R0	{2.28649, 15.9017}	{2.03017, 4.51841}	{{0.164552, 1.56924}, {3., 5.76361}}	{40.0005, 72.2238}	inf	132.	99.5907	{8.1327, 299.956}	77.78	0.525317	newly	68.	110.9	{10., 292.}	125.	0.425147	Reff	1.71487	0.361487	{0.0336205, 0.583455}	79.5333	ar	880.	1592.73	{791., 2095.}	88.0261
45	R0	{2.65121, 18.6339}	{2.538, 7.21683}	{{2.11596, 2.52426}, {3., 12.6588}}	{18.4786, 61.2704}	inf	87.	219.689	{68.0627, 377.616}	163.758	0.376232	newly	50.	211.91	{65., 526.}	329.38	0.524491	Reff	2.3092	0.78738	{0.228848, 1.51277}	65.9025	ar	928.	1554.26	{1086., 2181.}	69.931
46	R0	{2.52291, 18.0251}	{2.10276, 4.76206}	{{0.177691, 1.68421}, {3., 6.18925}}	{34.2413, 73.8921}	inf	129.	94.8743	{12.4888, 242.11}	72.0944	0.475963	newly	55.	102.69	{12., 279.}	132.382	0.368651	Reff	1.99814	0.387452	{0.034062, 0.598801}	80.8513	ar	894.	1572.04	{693., 1853.}	84.5615
47	R0	{2.22618, 17.1225}	{2.22331, 7.1307}	{{1.23958, 2.67108}, {3., 14.626}}	{33.4372, 60.3169}	inf	75.	157.367	{35.6163, 373.126}	133.784	0.30163	newly	39.	139.39	{28., 338.}	269.308	0.417354	Reff	1.91451	0.492104	{0.0970959, 1.23646}	75.3641	ar	850.	1628.35	{1105., 2070.}	92.6129
48	R0	{2.04405, 16.073}	{2.24582, 6.72079}	{{1.23239, 2.77521}, {3., 13.2457}}	{37.1206, 60.5785}	inf	64.	146.401	{29.5303, 354.783}	150.803	0.319048	newly	41.	137.87	{25., 329.}	252.171	0.393852	Reff	1.69452	0.479781	{0.0917593, 0.927994}	74.1917	ar	779.	1571.05	{1010., 1824.}	103.131
49	R0	{2.84396, 18.097}	{2.54892, 6.8343}	{{2.13501, 2.61386}, {3., 12.3463}}	{16.0803, 62.2352}	inf	134.	228.739	{54.3244, 435.52}	94.3754	0.295023	newly	69.	232.93	{61., 581.}	247.348	0.447654	Reff	2.18132	0.866581	{0.251377, 1.50793}	60.7255	ar	952.	1550.79	{1074., 2178.}	66.1439
50	R0	{1.85341, 18.0975}	{2.25875, 6.78487}	{{1.3233, 3.464}, {3., 12.0675}}	{42.1582, 62.9223}	inf	138.	161.272	{17.4296, 375.024}	84.0552	0.392002	newly	56.	149.69	{13., 402.}	198.946	0.437715	Reff	1.27329	0.498363	{0.0434156, 1.28649}	65.6657	ar	754.	1628.77	{1040., 2425.}	118.824
